# Digital health system for personalised COPD long-term management

**DOI:** 10.1186/s12911-017-0414-8

**Published:** 2017-02-20

**Authors:** Carmelo Velardo, Syed Ahmar Shah, Oliver Gibson, Gari Clifford, Carl Heneghan, Heather Rutter, Andrew Farmer, Lionel Tarassenko, on behalf of the EDGE COPD Team, Andreas Triantafyllidis, Veronika Williams, Maxine Hardinge, Christy Toms, Louise Jones, Stephanie Robinson, Jonathan Price, Linda Heritage

**Affiliations:** 1 Department of Engineering Science, University of Oxford, IBME, Oxford, UK; 2 Nuffield Department of Primary Care Health Sciences, University of Oxford, Oxford, UK

**Keywords:** COPD, Self-management, Digital health, Adaptive thresholds, Automatic alerts

## Abstract

**Background:**

Recent telehealth studies have demonstrated minor impact on patients affected by long-term conditions. The use of technology does not guarantee the compliance required for sustained collection of high-quality symptom and physiological data. Remote monitoring alone is not sufficient for successful disease management. A patient-centred design approach is needed in order to allow the personalisation of interventions and encourage the completion of daily self-management tasks.

**Methods:**

A digital health system was designed to support patients suffering from chronic obstructive pulmonary disease in self-managing their condition. The system includes a mobile application running on a consumer tablet personal computer and a secure backend server accessible to the health professionals in charge of patient management. The patient daily routine included the completion of an adaptive, electronic symptom diary on the tablet, and the measurement of oxygen saturation via a wireless pulse oximeter.

**Results:**

The design of the system was based on a patient-centred design approach, informed by patient workshops. One hundred and ten patients in the intervention arm of a randomised controlled trial were subsequently given the tablet computer and pulse oximeter for a 12-month period. Patients were encouraged, but not mandated, to use the digital health system daily. The average used was 6.0 times a week by all those who participated in the full trial. Three months after enrolment, patients were able to complete their symptom diary and oxygen saturation measurement in less than 1 m 40s (96% of symptom diaries). Custom algorithms, based on the self-monitoring data collected during the first 50 days of use, were developed to personalise alert thresholds.

**Conclusions:**

Strategies and tools aimed at refining a digital health intervention require iterative use to enable convergence on an optimal, usable design. ‘Continuous improvement’ allowed feedback from users to have an immediate impact on the design of the system (e.g., collection of quality data), resulting in high compliance with self-monitoring over a prolonged period of time (12-month). Health professionals were prompted by prioritisation algorithms to review patient data, which led to their regular use of the remote monitoring website throughout the trial.

**Trial registration:**

Trial registration: ISRCTN40367841. Registered 17/10/2012.

## Background

The term ‘digital health’ is used to encompass a wide range of technologies that include mHealth, telehealth, and connected health [1, 2]. In this paper, we specifically refer to the use of complex mobile health solutions for the remote monitoring and management of patients. In these scenarios, patients typically transmit their vital-sign data to healthcare professionals (HCP) in real-time. In most cases, the aim of the digital health system is to monitor the patient’s wellbeing remotely between visits to their clinician [3].

In recent years, many research groups have tried to design or adapt technical solutions for patients with chronic conditions [4–8], however, problems encountered include difficulties experienced by patients when using the technical solution, low compliance rates, and lack of personalisation (for example, restricting the use to specific times during the day) [9].

Similar observations have been reported in recent systematic reviews [4, 10, 11]. For example, O’Connor et al. [10] reviewed how patients and the public engage with and enrol in a broad range of digital health interventions. They recommended that digital health products and services should be tailored to lessen rather than increase the self-care burden of treatment. The aim should be to integrate digital health with patients’ current lifestyle, as a one-size fits all approach is unlikely to be effective.

Despite such limitations that may impact on usability, there have been a number of recent randomised controlled trials (RCTs) of telehealth interventions for the management of chronic conditions such as chronic obstructive pulmonary disease (COPD) [4–8, 12–15]. The three large-scale RCTs of long-term monitoring including people with COPD have found little or no substantial benefit for the participants [5–8], and have called for additional, better targeted RCTs [4]. Some smaller-scale trials have reported positive outcomes in terms of quality of life (QoL) improvement [14, 15], reduced risk of emergency department attendance and hospitalisation [13]. The Whole-System Demonstrator trial in the UK found little effect on the risk of death [5]. However, an evidence synthesis [16] suggested that there was an absence of high-quality evidence for management of COPD compared to other chronic conditions (e.g., heart failure, diabetes).

One of the main challenges to effective implementation of digital health systems is a lack of compliance with the use of technology. An extensive mixed-methods study [17], identified engagement from both patients and professionals as being key to the success of a telehealth intervention and noted the challenges faced by studies that had struggled to engage participants and clinicians.

In general, previous studies have shown that participants are receptive to the use of telemonitoring as a self-management approach. Generic telehealth literature reviews [11, 18] show an initial high level of acceptance and satisfaction with the telehealth systems employed in the studies considered, and, in several cases, there was a high level of compliance with self-monitoring.

However, even amongst well-designed studies, [11, 18] compliance tends to decrease over time, most rapidly in the period immediately after the beginning of the studies. Compliance beyond the initial period depended on the intervention type, on the condition treated, and on the characteristics of the patient population involved. Higher levels of compliance throughout the studies were associated with the following characteristics: low-burden and short interventions, type of chronic condition (for example, higher compliance with heart failure patients) and the age of the patients. The authors of both reviews identify this decrease in adherence over time as a critical issue to be addressed and further investigated, as chronic illnesses require long-term monitoring.

The literature on compliance with telehealth interventions points to the importance of integrating components into the intervention that promote both adoption and retention. Solutions proposed in the literature include: conveying information correctly and appropriately, lowering the entry barrier by softening the perception that some patients have of telehealth systems as being too ‘difficult’ to use, and enhancing the sensation of empowerment that patients feel when using effective telehealth solutions [11, 18–20]. If those conditions are met, the patients are more likely to engage and maintain long term compliance with the system.

Considerations of compliance led us to three observations that underpin the design of our digital health system and its subsequent use in clinical studies with COPD patients [21]: (i) the use of remote monitoring on its own may not be sufficient to guarantee the success of a telehealth/digital health intervention. An effective digital health strategy must also include a self-management component; (ii) one-size-fits-all approaches to both the design of the mobile applications and the alerting algorithms are prone to failure since they do not take into account natural variations in patient physiology or behaviour and (iii) state-of-the-art technology (based on the convergence of communications and computing) should be the basis for modern, scalable digital health platforms. These observations are aligned with the conclusions of several papers and systematic reviews on the topic of telehealth applied to the management of chronic conditions [4, 10–12, 17, 19, 20, 22]. Based on these observations, the digital health system that we designed includes a self-management component as well as self-monitoring, and was personalised as much as possible to the patient’s needs.

Gregersen et al. [4] call for more research on the evaluation of digital health systems through large-scale controlled trials. It was therefore our intention to evaluate our novel digital health solution at scale in the context of a randomised controlled trial, provided that preliminary testing with patients was successful.

The system was initially tested in a pilot study with 23 COPD patients [23]. The results of this pilot study were sufficiently positive to justify the design and implementation of an RCT using 2:1 allocation to examine experience with the intervention in detail. Both the pilot study and the RCT were part of the sElf-management anD support proGrammE (EDGE) project [21].

In this paper, we discuss the potential for patient-centred design to achieve highly usable and effective digital health applications for self-monitoring and self-management; we describe the strategies which we developed during the pilot study to achieve high-quality vital-sign recording in a home setting; and we present the results on usage of the system by patients in the RCT, analysing in detail the data recorded by the 110 patients who used the EDGE system for the 12 months of the trial. The analysis of the primary and secondary outcomes of the RCT is presented in a parallel clinical paper (Farmer AJ, Williams V, Velardo C, Shah SA, Yu L-M, Rutter H, et al.: Self-management support using an Internet-linked tablet computer compared with usual care for chronic obstructive pulmonary disease: results of a randomised controlled trial, submitted).

## Methods

This section describes the following: (a) the EDGE digital health system; (b) the methodology employed during its design; (c) the RCT in which the system was deployed; and (d) the data analysis phase.

### System architecture

**Fig. 1:**
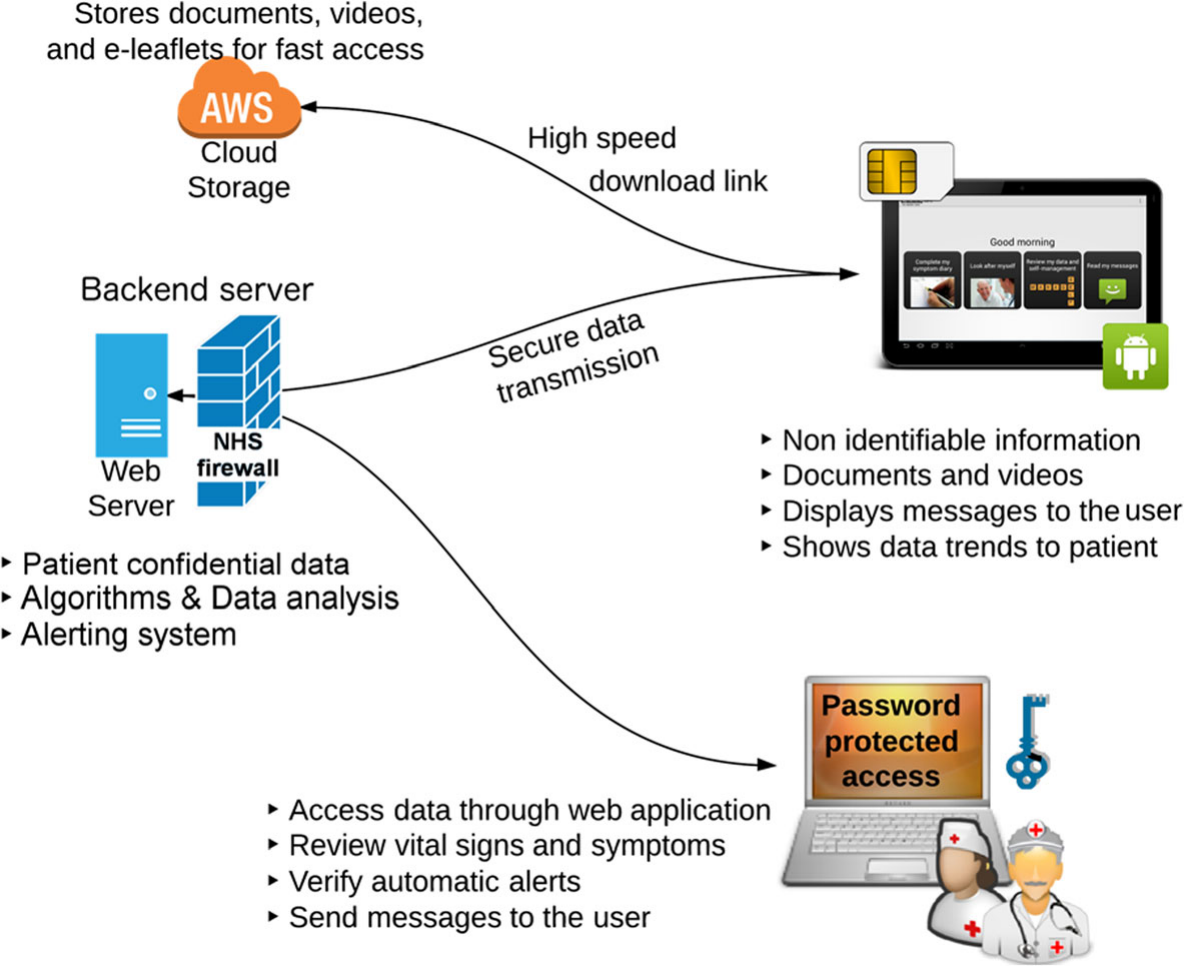
System overview: the Android mobile application sends data to a secure web server within the NHS network where data are stored. The data can be screened by the clinical team through a web application

The system architecture for the EDGE digital health system (Fig. 1) comprises a general-purpose tablet computer running on the Android operating system, a backend server within the local National Health Service (NHS) network, and a third-party server (Amazon S3).

A mobile application (“the application”) was developed to run on the tablet computer: it collected self-reported questionnaire and pulse oximeter data from the patient, and synchronised them stripped of personal identifiers automatically with the NHS server (backend system), for security reasons the tablet did not contain any personal information (e.g., name or date of birth). Each information exchange was based on pseudonymised data transmitted over a secure link, a tablet would be identified by an identifier generated on the server at the moment of the configuration. Data were stored in the backend server hosted within the local NHS network, behind its firewall. Here data storage and data analysis tasks ran in parallel to summarise and report the status of each patient to the healthcare professionals. The clinical and technical teams could interact with the system to configure new kits, monitor data from a web application customised for the task, and effectively communicate with the patient through text messages. Access was granted only to the clinical team that participated to the study and to researchers covered by NHS Research Passport.^1^ Finally, a high-speed download link was provided by a third-party server (Amazon S3) in order to distribute videos and documents to be downloaded by the Android application so as to provide self-management information, personalised to each patient’s needs. The third-party service was chosen because it provided us with larger bandwidth than the NHS link, but no patient data were ever stored on the third-party server.

We decided to use a backend server inside the local NHS network protected by its tightly-controlled security firewall to ensure a high level of data security. At the same time, the mobile application did not contain nor transferred any personal information which could identify the patient to the server. All communications used a Transport Layer Security certificate signed from certification authority (DigiCert Inc.), and an electronic signature validated each connection before data were accepted and stored in the backend server. The server was equipped with technologies (e.g., Matlab and Python) to enable the design of algorithms for automatic data quality control, signal analysis, and data reporting.

The patients’ use of the application (buttons clicked, sections visited, time of use) was monitored in the background via an internal logger and usage data, in addition to the vital sign values and answers to the patient diary questions, were uploaded to the backend server. A similar log system was employed on the backend server to monitor nurse access to the web portal.

### Co-design of the EDGE digital health system

A usability assessment, talk-aloud session was conducted with a group of 10 patients to test a prototype version of the patient application on both a 7” and on a 10.1” computer tablets. The bigger tablet PC was preferred by patients to the smaller version because it provided a much larger display, with the possibility of displaying text with larger fonts, and because it gave a better handheld experience.

To enhance usability we included the following: a simple interface (no keyboard); videos and other multimedia content to facilitate conveying information correctly and appropriately; and user-friendly procedures (supported by signal-quality algorithms) to ensure the collection of high-quality data, thus lowering the technical barriers for the competent use of the system. Instead of a bespoke tele-health box, we employed an Android tablet with Bluetooth communication to an external oximeter.

For the pilot phase, each kit was therefore composed of an Android tablet PC (Samsung Galaxy Tab 2) and a finger pulse oximeter (Nonin 9560 Onyx). The equipment was handed out by a research nurse, together with a leaflet that explained the basic maintenance of the tablet PC (turning on/off and recharge) and of the pulse oximeter (changing batteries).

The application design, especially the self-management module, required a careful analysis of patients’ needs. Consequently, feedback from the clinical collaborators and the patients was crucial in the design of the application. Our design process, analogous to our previous work [24], made sure that the patients’ feedback was collected during two co-design workshops (attended by focus groups of COPD patients), and during several home visits (when research engineers and/or research nurses met patients for prototype evaluation). Patients’ feedback was then discussed during weekly or monthly meetings between the healthcare professionals and the technical team, so that appropriate changes to the digital health system were implemented and tested as soon as possible. The result of these iterations (Appendix Figure 7) led to an easy-to-use and intuitive interface for the application that needed no additional information (e.g., operation manual) to explain how to use its functionalities.

Twenty-three patients with different COPD severity levels were recruited to help in the design of the pilot study, during which multiple features were designed and tested before finalising the version of the application for the randomised controlled trial phase. The research team agreed through consensus and guidance by clinical experts which features to incorporate in the EDGE system, following a ‘continuous improvement’ approach.

### Use of EDGE digital health solution in RCT

The randomised controlled trial involved 166 COPD patients (56 in the usual care group, 110 in the digital health intervention group, Appendix Table 1). The progress of the trial was reviewed by the research team in monthly meetings where feedback from patients, carers, and the research nurses was discussed.

### Data collection and data quality

One of the main advantages of digital health, compared to conventional care, is the possibility of administering questionnaires systematically, and collecting health-related data remotely and directly from the patient while they are in their home environment. Therefore, one of the main non-functional requirements of such solutions should be the reliability of the data collected [25]. Especially when designing self-management modules, digital health system designers have to ensure that all measures have been employed to guarantee reliable data collection.

**Fig. 2:**
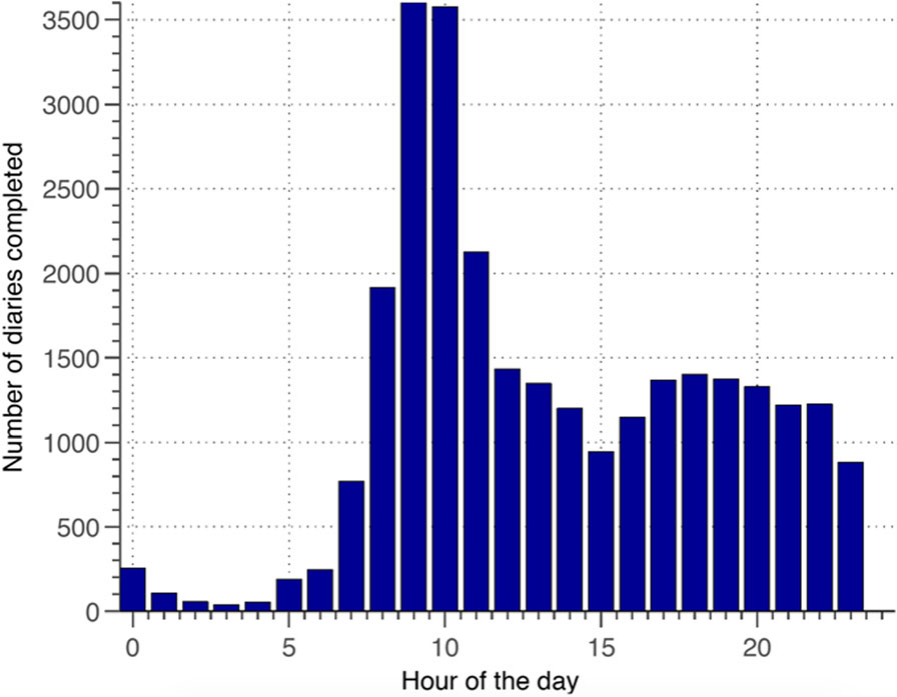
Time of use of the EDGE application by the intervention group participants during the RCT. The majority of patient diary sessions took place in the morning or afternoon, but a non-negligible number of diaries were completed at night

With the EDGE system, participants were asked to record on the computer tablet their answers to the symptom questions daily, and their answers to the anxiety and depression questions monthly. The application did not mandate patients to complete their diary at a specific time of the day, in order to allow for greater flexibility and the freedom to choose a time that suited their daily routine (Fig. 2). To allow the clinical team to track participants ‘emotional well-being’, anxiety and depression questionnaires, based on a combination of PHQ-2, GAD-2, PHQ-8, and GAD-7 questionnaires [26, 27], each had to be answered monthly. The score computed from each questionnaire was used to select a set of videos to be proposed to the patient at the end of the data-entry session. The number of questions asked was adjusted according to pre-defined flow diagrams and the answers given to the questions by the patient. If the answers indicated a major increase in anxiety and depression, then that patient was flagged to his or her General Practitioner.

As part of the daily routine, each patient also had to record pulse oximetry data for 30 s (generating pulse rate and oxygen saturation data at a rate of 3Hz). There is recent evidence that oxygen saturation readings from a pulse oximeter heighten patients’ awareness of their condition and gives them confidence to make improved self-management decisions [28]. The pulse rate and oxygen saturation measurements were transferred automatically via Bluetooth by the pulse oximeter to the tablet, together with the entire photoplethysmographic signal (the cardiac-synchronous waveform recorded by the pulse oximeter, here at a rate of 75 Hz).

Patients were introduced to the use of their kit by a community respiratory nurse at the first visit to their home after being allocated to the intervention group in the RCT and were asked to use their tablet computer on a daily basis at a time that would suit their daily routine.

The application design was divided into three modules. The data collection module allowed the patient to record their answers to simple questions in the patient diary and also their pulse oximetry data (pulse rate and oxygen saturation values) simply by placing their finger in the pulse oximeter probe. The communication module ensured that the clinical team could send messages to the patient (e.g., a message from the nurse to help set up a home visit, or a message advising a visit to the general practitioner (GP)). Lastly, the self-management module offered patients feedback about their physical well-being (through the use of graphs of their vital sign values and of their answers to the symptom diary questions), motivational and psychological support (e.g., through the use of videos demonstrating respiration techniques, or helping to manage anxiety and mood related issues).

The content of the self-management section was remotely configured for each patient by the clinical team and the content could be personalised to match the current profile of the patient (e.g., back-up medication information, oxygen use, and smoking cessation information).

Although the positive aspects of digital health such as the possibility of daily monitoring, personalised care delivery, and reduction of unwanted biasing effects (e.g., the “white coat effect” in the case of blood pressure measurements), home monitoring systems require patients to report their symptoms and record their vital signs with no help. The lack of specialised support might increase the chances of human error in recording the answers to the symptom diary questions and using the medical equipment (here the pulse oximeter). Therefore, appropriate techniques for data quality check were considered during the design of the EDGE digital health intervention. Several solutions for both symptom reporting and vital sign recording were employed to improve the reliability of data collection.

For symptom self-reporting we employed a ‘select and continue’ approach that allowed patients to ‘select’ their answer and then ‘continue’ with the next question/symptom, thus allowing them the time to change their mind as many times as they deemed necessary before continuing.

To ensure that the Bluetooth-enabled pulse oximeter was used effectively and generated high-quality data, explanatory text, images, and videos were assembled to show how to take a reading. Moreover, a feedback mechanism alerted the patient when data of poor quality were being recorded. For example, a message prompted patients to sit with their hand still, their wrist flat on a surface, and with their finger in the pulse oximeter for the whole duration of the oxygen saturation recording (30 s).

The signal quality index provided by the manufacturer (Nonin Inc.) was put to good effect. If poor signal quality was detected, the EDGE application would extend the recording time for a further10 s. If this failed to provide sufficiently high quality data (a continuous recording with no artefact for 30 s), patients would be presented with the option to attempt recording a second time. Recording would not be attempted a third time; the data would be accepted as ‘best effort’, not displayed to the patient, but still transmitted to the server for research purposes alongside the signal quality information.

### Self-management and exacerbation management

Previous versions of digital health systems have followed a paradigm of regular clinical review of all patient data to allow clinicians to exercise clinical judgment about the need for clinical intervention. The approach we undertook in the EDGE project was to recognise that patients are not in need of attention when they are well. We hypothesised that data could be obtained during the study to help define patients as either requiring, or not requiring, nurse review. Patients recognised as requiring the immediate attention of the respiratory nurse were prioritised for urgent follow-up, and their profile reviewed, Priority was assigned according to the number of ‘alerts’ in the most recent two weeks since the last review. In this context alerts were generated when the vital sign value (pulse rate or oxygen saturation) went above (or below) a certain threshold or the overall symptom score (computed out of nine questions [23]) went above a certain threshold.

Since the physiology of each patient is different, a robust alerting mechanism needs to take into account intra- and inter-person variability. Telehealth systems have tended to rely on fixed thresholds, or values manually tuned by health care professionals [5–8]. We relied instead on signal processing to provide automated, data-driven, personalised alert thresholds. A training period of 40 data points (corresponding approximately to the first six weeks of system usage) was adopted. The training period was chosen in collaboration with a panel of experts and selected in order to allow time for the patient to become familiar with the system, and for sufficient symptom and vital sign data to be collected to characterise that patient.

**Fig. 3:**
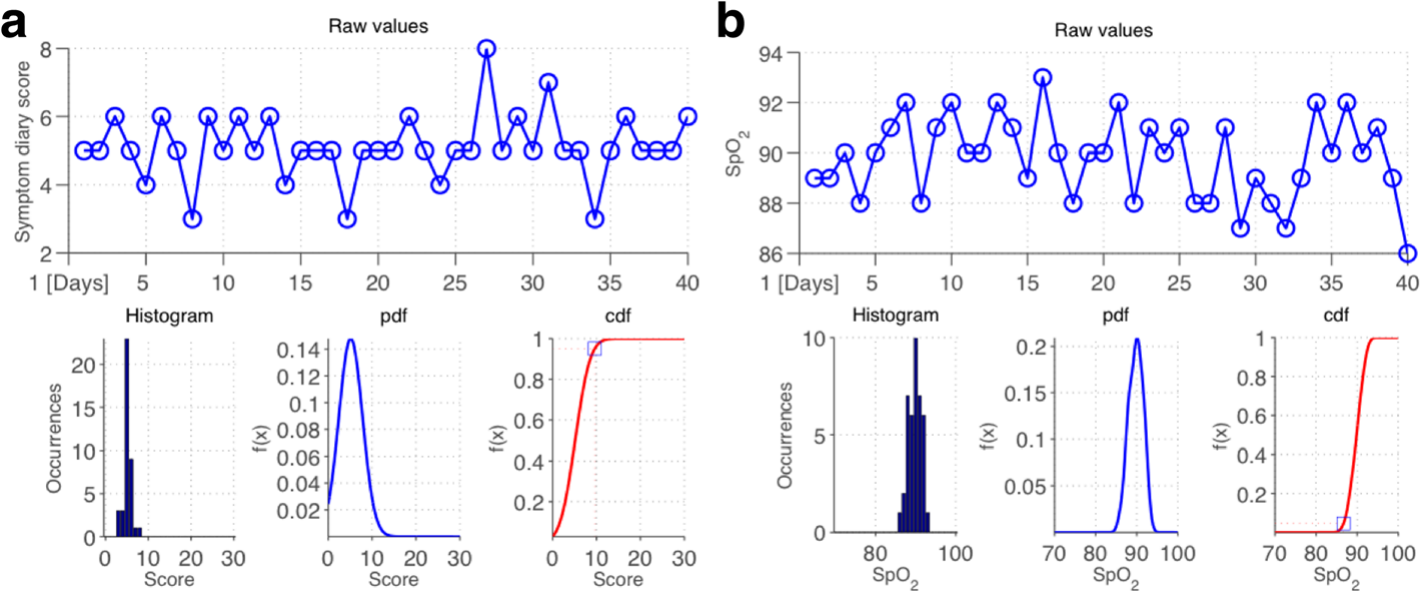
An illustration of how the alerting thresholds were computed for the symptom diary score (﻿**a**) and SpO2 data (**b**) from one patient. From the top anti-clockwise: (1) raw data points, (2) distribution of the data points, (3) probability density function (PDF), (4) cumulative density function (CDF). The threshold value is indicated by the small square in the last plot and it is equal to the value corresponding to the 95^th^ percentile (5^th^ in the case of SpO2). Gaussian kernels were used instead of rectangular ones in order to achieve a smooth PDF and CDF

A univariate approach was chosen at this stage to analyse the vital sign and symptom score values separately. From the data acquired during the training period, we computed the alerting threshold as the value corresponding to the 95^th^ percentile for that parameter (see Fig. 3). By using this approach for oxygen saturation, pulse rate, and overall symptom score, we were able to compute personalised alert thresholds for each patient.

We implemented this approach during the pilot study to test its viability with the healthcare professionals responsible for managing the patients and we then used it during the randomised controlled trial to enable alerting thresholds to be configured automatically for each patient.

Treating each parameter separately makes the alerting system easy to understand for the respiratory nurses reviewing the patient data and straightforward to implement whilst allowing personalisation to the patient’s data.

## Results

In a parallel clinical paper, we present an analysis of the primary and secondary outcomes of the RCT (Farmer AJ, Williams V, Velardo C, Shah SA, Yu L-M, Rutter H, et al.: Self-management support using an Internet-linked tablet computer compared with usual care for chronic obstructive pulmonary disease: results of a randomised controlled trial, submitted). Here we focus on the technical aspects of the use of our system during the RCT: system usage for the intervention arm of the RCT (tablet computer usage for self-monitoring and self-management by patients, and study website usage for remote patient management by respiratory nurses), and vital-sign data analysis for personalised alerting. We report on the data collected by the system, and review the performance of the algorithms applied to the patient data collected by the intervention group during the RCT.

### Analysis of system usage in the RCT

One hundred and ten participants were involved for a 12-month period: data were included from all participants allocated to the intervention arm regardless of the extent to which they used the system.

The time of day chosen to use the application varied substantially across all patients, with a majority preferring the morning, while some preferred the evening or late evening to record their symptoms and check the value of their oxygen saturation.

Most patients completed their diaries within 2 h of their chosen time (Appendix Figure 10). This confirmed our assumption that, given enough flexibility, patients will develop a habit of use, thus increasing compliance.

**Fig. 4:**
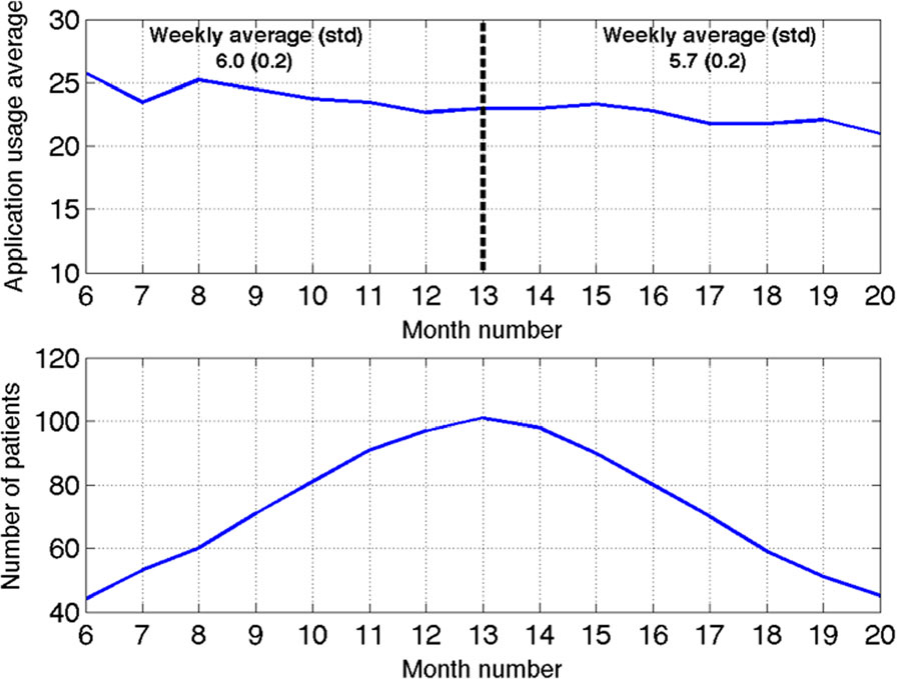
The plot shows the patient compliance with the mobile application across the study. Compliance (measured as number of days of usage per week) remained stable throughout the study. The first 5 and last 4 months of the RCT were not included in the calculation as they included too few participants to compute meaningful values

Patient compliance was defined as the number of days a week that a patient used the system (so maximum compliance could only be as high as 7). Overall compliance was then defined as the compliance averaged across all patients. In Fig. 4 we show the trend of system compliance for all intervention group patients. The value is stable throughout the RCT, with an average (sd) of 6.0 (0.2) days a week for months 6 to 13 and an average (sd) of 5.7 (0.2) days a week for months 13 to 20.

**Fig. 5:**
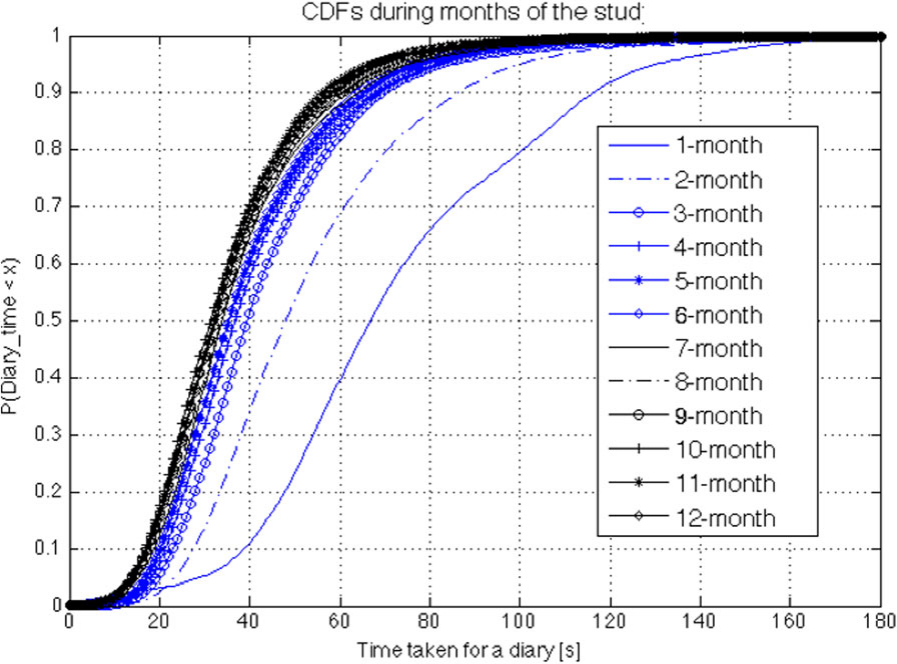
Learning curve for use of the digital health application: each line represents the cumulative distribution function (CDF) for the time needed by each patient to complete the symptom diary, for the i^th^ - month. Over time, patients become quicker at completing their symptom diary

The learning curve associated with the use of a new digital health system also influences the user experience. We have used the time needed to complete the symptom diary as a proxy to measure the progress of patients. The data from the interaction log were analysed and the completion time for each symptom diary was computed. We grouped the data according to the time since the beginning of the study, and computed twelve cumulative distribution functions (CDF), one for each month spent in the study, as shown in Fig. 5. The closer a CDF gets to the y-axis, the shorter the completion time. The plot shows that patients become quicker at using the system during the first three months of use. The majority of symptom questionnaires (96%) were completed in less than 1m40s after the third month of use.

The web portal (Appendix Figures 8 and 9) also monitored how the nurse used the system. Over the course of the trial the nurse accessed the web interface, on average, 33 times ± 15 (range: 11–79) per patient, used the system to track notes about patients on average 32 times ± 16 (range: 3–79), and changed the medication plan 8 times ± 4 (range: 2–20) per patient. Since each participant was in the trial for 12 months, the equivalent figures are 2.75, 2.67 and 0.67 times per month. The two research nurses found this workload to be less than that associated with conventional care. The system gave the possibility to the research nurse reviewing the patients’ data during the RCT to dismiss alerts by accomplishing one of a set of predefined actions on the patient’s profile. The actions were designed to map the workflow of the community nurse team: a) Symptom diary reviewed - no action required, b) GP/Practice nurse contacted and advised to contact patient, c) Respiratory nurse contacted and advised to contact patient, d) Community respiratory nurse contacted and symptom diary details handed over, e) Patient contacted by the research nurse (with details about the reason), f) Letter sent to GP after GAD/PHQ diary alert, g) Sign-off (Addition of a visible tick mark on the patient’s profile, which remained for the next three days).

In case of action (e) and (g) the alerts were then disabled for three consecutive days.

Of all actions, the first one (a) was initiated by the nurse a total of 5,348 times (48 times per participant, i.e., four times per month, or once a week), usually in combination with action (g), which temporarily disabled alerts (3,458 times, 31 times per participant). Other actions were undertaken far more rarely: (f) 36 times, (e) 35 times, (b) 7 times, (c) 4 times, and (d) 1 time. These data provide clues as to which actions could be grouped together in the next version of the digital health system, and which ones to remove.

The health care professionals using the system disabled an average (sd) of 42 (35) alerts per patient. When clustering alerts which occurred within a 7-day window, the number of disabled alerts decreases to an average (sd) of 20 (11) weeks per patient.

### Data quality

Both algorithmic and design features enabled the system to collect and retain good quality data despite the unconstrained, home-based use case. As a result, we collected 28,184 data sessions containing both symptom diary and oximetry data, plus an extra 3508 sessions with symptom diary only.^2^ Out of the 28,184 sessions, 26,293 (93.3%) were of high quality and only 1,891 sessions (6.7%) were deemed to be of low quality (due to motion artefacts).^3^ As patients recorded their vital signs after very little training, these results are very promising and indicate that high-quality data collection should be possible in large-scale digital health systems.

### Vital-sign data analysis

**Fig. 6:**
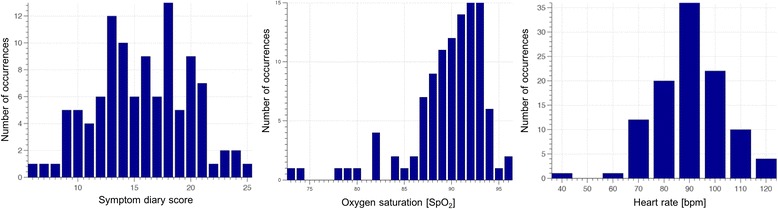
Distributions of alert thresholds calculated after run-in period for each of the 110 intervention-group patients, for the different data collected: symptom diary score (left histogram), oxygen saturation (center histogram), and heart rate (right histogram)

Using the personalised univariate approach developed during the pilot study allowed us to handle intra-patient changes automatically. By selecting the threshold based on the 95^th^ percentile of the data distribution during the run-in period, we were able to obtain a similar alert rate (5%) for most patients (those whose physiology did not change after the run-in period). To demonstrate the large variability in patient physiology, Fig. 6 shows the histograms of the threshold values computed by our system for the 110 intervention-group patients in the RCT. The figure indicates that using a single value for the alert threshold for all patients is sub-optimal; for some patients, the real alerts would be missed in the midst of a plethora of false alerts, while for others the same fixed threshold would lead to alerts being missed.

In order to cope with intra-patient variability over time, the EDGE digital health system could make the system re-compute automatically a patient’s alert threshold for a patient whenever the baseline values for that parameter changed significantly. This functionality was used for each patient an average of 4.1 times (±1.3) during the study, taking into account the fact that there are three threshold values: one for oxygen saturation, one for heart rate and once for the overall symptom score.

## Discussion

Previous trials have tended to explore the use of telehealth for long-term self-monitoring using static bespoke equipment, limited patient engagement strategies, and without offering personalisation of either data collection or feedback of data [5–8, 10]. The shortcomings of these systems have been well rehearsed (for example, see the paper on the TELESCOT trial reviewed in the Background section at the start of this paper). It is difficult for us to compare our system with previous telehealth systems which have mostly used dedicated hardware systems. The novelty of our approach lies in the combination of an easy-to-use application on a *standard computer tablet* (Samsung Galaxy Tab 2), promotion of self-management using videos, patient-centred design, and personalised alerting. We have shown that this approach led to very high compliance figures, sustained throughout a 12-month period, Most telehealth studies do not report compliance rates for the intervention group patients (except for self-monitoring of blood glucose in diabetes) but a recent systematic review of home telemonitoring in COPD [9] reported difficulties with use of technology, which in a number of studies led to low compliance rates.

We have presented the design and development of the EDGE COPD system and the results of the evaluation of its use both by patients in the intervention group and for remote management by respiratory nurses during a 12-month RCT. Patients were provided with a personalised application running on an Internet-enabled computer tablet, providing mobility to those who wanted to carry on their monitoring while away from home (e.g., when at the hospital or in a different house). Additionally, the system allowed the recording of self-monitoring data at any time, thus encouraging the patient to find the appropriate time that fitted their daily routine. The data collection process allowed patients to record high-quality physiological data, with only 6% of the photoplethysmographic (pulse oximeter) waveforms affected by motion artefacts.

The work presented here shows the benefits of our patient-centred approach:

1. enhanced patient compliance, as demonstrated in the “Analysis of system usage in the RCT” section;
2. improved ease of use and collection of high-quality data (see the “Data collection and data quality” section);
3. value of adaptive algorithms based on individual patient physiology, as discussed in the “Self-management and exacerbation management” and “Vital-sign data analysis” sections.

Compliance rate and usage-time demonstrate that the mobile application is easy to use and show that patients become proficient in completing the symptom diary within three months.

The compliance measure remained stable throughout the study, reaching an average of 6.0 times per week per patient. Usage of the system was consistently high throughout the duration of the study regardless of age or gender. With only 110 participants in the intervention group, it is not possible to carry out a quantitative analysis of the usage data according to gender, age, COPD severity or previous computer usage. We have qualitative evidence [29] however that the system was useful and acceptable for all the types of patients in our study, regardless of their level of familiarity with computers.

The data collected demonstrate how the clinical team engaged with the management website and with the patient through the functionalities provided by the EDGE back-end system.

We developed a univariate statistical approach to alert personalisation which was used to provide immediate feedback to the clinical team, and could also provide a baseline for future attempts at improved algorithms for the detection of exacerbations [30, 31]. Results from the qualitative study [29] and from the analysis of the RCT results (Farmer AJ, Williams V, Velardo C, Shah SA, Yu L-M, Rutter H, et al.: Self-management support using an Internet-linked tablet computer compared with usual care for chronic obstructive pulmonary disease: results of a randomised controlled trial, submitted) suggest that patients are comfortable with using the digital health intervention to self-manage their condition, and that the use of the system may help in reducing the usage of healthcare resources (practice nurse and GP visits). Initial results from usability assessment of the website also confirm high levels of system usability and technology acceptance from a wide range of professions from the community respiratory nurses and physiotherapists who interact with COPD patients on a regular basis.

A limitation of this paper is that we are not reporting the impact of our digital health system on outcomes (for example, the frequency of use of primary care) as these results are presented in a parallel clinical paper. A further limitation is the fact that our sample size did not allow us to explore the effect of gender, age, COPD severity or familiarity with computer technology on the acceptability and the usage of the system; however, the qualitative study reported in [29] indicated a positive attitude towards the usability of the EDGE system. Finally, we were not able to carry out an investigation of the cost-effectiveness of our system. The use of computer tablets as devices to access the internet continues to increase [32]. Future studies will be able to benefit from using the devices owned by participants, with sample sizes such as to make the reliable assessment of cost effectiveness feasible.

## Conclusion

Usage data for the trial confirm that patient-centred design and iterative strategies aimed at refining digital health platforms (e.g., continuous integration) enable the development of an easy-to-use interface, which promotes compliance in COPD patients. Long-term, sustained use of the digital health platform was shown to be feasible and acceptable to participants. Patients reported their daily symptoms, medication use, and were able to measured physiological variables, generating a large dataset of high-quality data. The healthcare professionals engaged closely with the system as demonstrated by their regular use of the website for remote monitoring of patients throughout the trial. We also demonstrated an effective way of integrating tools for the generation of personalised alert thresholds in a large population of individuals. The high levels of usability and compliance suggest that the platform is ready for translation into standard care. Following the end of the RCT, the EDGE-COPD system has been adopted by a number of respiratory nurses in Oxfordshire to manage patients remotely.

## Figures and Tables

**Fig. 7:**
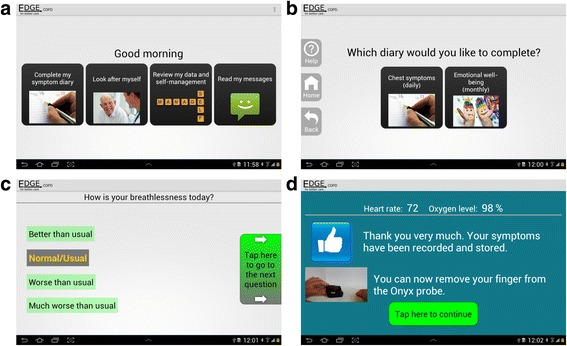
Samples of the user interface of the Android mobile application: **a** main menu, **b** symptom questionnaire selection, **c** questionnaire data entry, **d** oxygen saturation measurement session summary

**Fig. 8:**
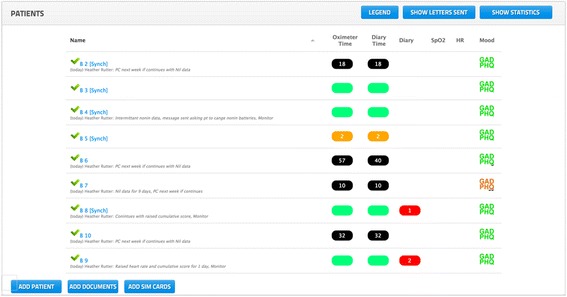
Main page on the back-end server with the patient list, the number of days since the last measurements or symptom diary session, and any alerts generated by the data collected through the Android mobile application

**Fig. 9:**
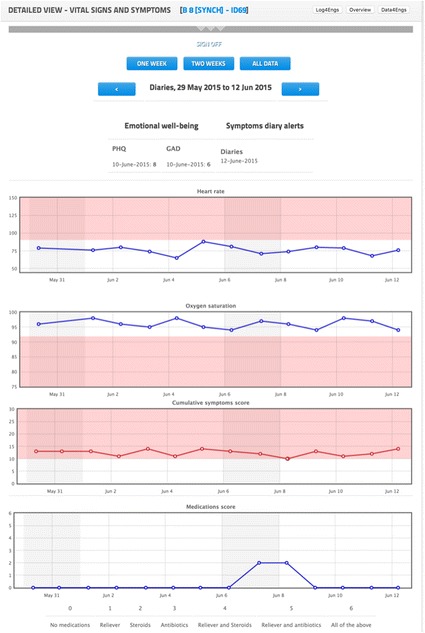
Individual patient page from back-end server, with details of the last 2-week period. In this case the overall symptom score was over the alerting threshold. The patient responded with a dose of steroids for two days (7–8 June)

**Fig. 10:**
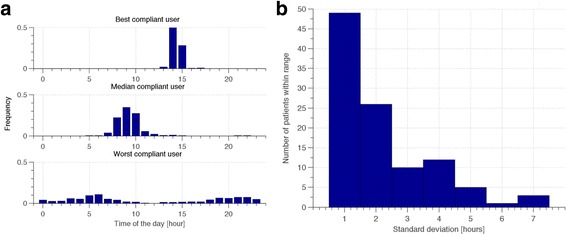
**a** Time of usage distributions. From top to bottom: Most compliant user (lowest standard deviation), median compliant user, least compliant user (largest standard deviation). **b** Distribution of standard deviations for usage time by intervention-group participants in RCT. The majority of participants (74) completed their daily session with less than 2 h standard deviation from their usual completion time

## Appendix

**Table 1:** Patient characteristics for the cohort of 110 patients in the intervention group that completed the 12-month randomised controlled trial

	Percentage
Age	40–50 year: 1.8%
	51–60 year: 16.5%
	61–70 year: 27.5%
	71–80 year: 44.9%
	>80 year : 9.1%
Gender	Male 62% (68)
	Female 38% (42)
COPD severity	Gold 1: 0%
	Gold 2: 37%
	Gold 3: 46%
	Gold 4: 17%
MRC dyspnoea scale [33]	MRC 1: 0%
	MRC 2: 16%
	MRC 3: 67%
	MRC 4: 17%

## Abbreviations

CDF: Cumulative distribution function
COPD: Chronic obstructive pulmonary disease
EDGE: sElf-management anD support proGrammE
GP: General practitioner
HCP: Health care professional
NHS: National health service
PC: Personal computer
RCT: Randomised controlled trial

## Footnotes

1
http://www.nihr.ac.uk/about-us/CCF/policy-and-standards/research-passports.htm - last accessed: 13 February 2017

2 The section of the app will record a symptom diary but not an oxygen saturation session in case of problems with the oximeter (low battery, problems with Bluetooth connection, participants deliberately removing their finger).

3 Low-quality data were not displayed on the website.
